# 1-Hour postprandial glucose target of < 120 mg/dL is superior to < 140 mg/dL in the treatment for gestational diabetes mellitus in relation to pregnancy outcomes: A retrospective study

**DOI:** 10.1007/s00592-020-01655-w

**Published:** 2021-02-12

**Authors:** Monika Żurawska-Kliś, Klaudia Czarnik, Szymon Szymczak, Marzena Wójcik, Katarzyna Cypryk

**Affiliations:** 1 Department of Internal Diseases and Diabetology, Medical University of Lodz, 251 Pomorska str., 92-213 Lodz, Poland; 2 Department of Structural Biology, Medical University of Lodz, 7/9 Żeligowskiego str., 90-752 Lodz, Poland

**Keywords:** Gestational diabetes mellitus (GDM), 1-Hour postprandial glucose (1-h PPG), Pregnancy outcomes

## Introduction

Maternal hyperglycemia during pregnancy is associated with short- and long-term adverse effects affecting both mothers and the offspring. Despite behavioral and pharmacological treatment, the rate of maternal and neonatal complications in pregnancies complicated with gestational diabetes mellitus (GDM) is still higher than in general population [1, 2]. Data from the Hyperglycemia and Adverse Pregnancy Outcome Study (HAPO) that was first published in 2008 showed a continuous positive correlation between glycemia on the 75-g oral glucose tolerance test (OGTT) and the rate of large for gestational age infants (LGA), neonatal hypoglycemia, cord blood C-peptide concentration > 90th percentile, and cesarean section in a cohort of approximately 25,000 pregnant women from different populations [3]. On this basis, the new diagnostic criteria for GDM were proposed and then incorporated into the most recommendations and standards, including these published by the World Health Organization (WHO).

Still, there is no universally accepted consensus on glycemic goals in the treatment for GDM because data on the effect of different glycemic targets on pregnancy outcomes in GDM are limited. Most scientific societies suggest the thresholds for glucose concentrations of 95 mg/dL at fasting and 140 mg/dL at 1-h postprandial (strength-of-recommendation grade B) [4].

In 2017, the Polish Diabetes Association (PDA) raised the 1-h postprandial glucose (1-h PPG) threshold from < 120 mg/dL to < 140 mg/dL [5]. This change prompted us to compare the obstetric results of women with GDM in two time intervals: treated using the previous (1-h PPG < 120 mg/dL) and present (1-h PPG < 140 mg/dL), less stringent, recommendations.

## Aims

The aim of this study was to examine whether the change of 1-h PPG target from < 120 mg/dL to < 140 mg/dL impacted the rate of adverse pregnancy outcomes in GDM.

## Methods

Medical records of 438 Caucasian pregnant women with GDM diagnosed at 17–28 weeks of gestation (the WHO 2013 criteria), who were treated at the Outpatient Department of Diabetology, Lodz, Poland, from 2014 to 2020, were retrospectively reviewed. Women aged < 18 or > 40 years with multiple pregnancy or with pregnancy after in vitro fertilization were excluded from the study. All participants were treated according to the PDA guidelines and received standard diabetes self-management counseling. There were 155 patients in Group A (target 1-h PPG < 120 mg/dL) and 238 patients in Group B (target 1-h PPG < 140 mg/dL) During the observation period, no changes to treatment standards were made other than the change of the target of blood glucose level at 1 h after meal. Baseline data on maternal characteristics and pregnancy outcomes were taken into statistical analyses. Statistical significance was set at the *p* < 0.05 level. Statistical analyses were performed using Statistica 13.1 software (Statsoft Polska, Krakow, Poland).

## Results

Baseline maternal characteristics and perinatal outcomes are shown in Table 1. Differences between groups were tested using the Mann–Whitney *U* test for continuous variables and Chi-squared test for categorical variables.

**Table 1:** Maternal clinical characteristics and pregnancy outcomes according to 1-h PPG goals

Variables	Postprandial goals		*p* value
	1-h PPG < 120 mg/dL (< 6.7 mmol/l) GROUP A (*n* = 155)	1-h PPG < 140 mg/dL (< 7.8 mmol/l) GROUP B (*n* = 238)	
*Maternal clinical characteristics*			
Median age, years (IQR)	28.00 (27.00, 30.00)	31.00 (28.00, 36.00)	**0.0000**
Median prepregnancy BMI, kg/m^2^ (IQR)	25.59 (22.58, 28.82)	25.00 (21.79, 29.00)	0.5016
Median FPG in OGTT, mg/dL (IQR)	86 (80, 96)	91 (84, 98)	**0.0028**
Median 1-h OGTT, mg/dL (IQR)	179 (159, 193)	180 (157, 198)	0.5139
Median 2-h OGTT, mg/dL (IQR)	156 (145, 165)	155 (132, 168)	0.5007
GDM diagnosis, gestational week (IQR)	26 (24, 27)	25 (18, 26)	**0.0001**
Insulin treatment during pregnancy yes, *n* (%)	66 (42.58)	114 (41.91)	0.8929
*Pregnancy outcomes*			
Natural birth yes, *n* (%)	68 (43.87)	119 (50.00)	0.2198
Cesarean section yes, *n* (%)	87 (56.13)	119 (50.00)	
Median gestational age at delivery, gestation week (IQR)	39.00 (38.00, 40.00)	39.00 (38.00, 40.00)	0.1254
Premature birth < 37th gestational week, *n* (%)	23 (14.84)	25 (10.71)	0.5650
Median neonatal birth weight, g (IQR)	3380 (3010, 3590)	3350 (3090, 3650)	0.6593
Macrosomia (> 4000 g) yes, *n* (%)	6 (3.87)	28 (9.89)	**0.0243**
LGA yes, *n* (%)	6 (3.87)	48 (16.61)	**0.00001**
SGA yes, *n* (%)	13 (8.39)	31 (10.73)	0.4316

Results are presented as median and interquartile range (IQR) for continuous variables or *n* (%) for categorical variables. Values in bold type are statistically significant at *p* < 0.05

*1-h PPG* postprandial glucose at 1 h after meals, *BMI* body mass index, *FPG* fasting plasma glucose, *OGTT* oral glucose tolerance test, *LGA* large for gestational age, *SGA* small for gestational age

The patients from the group A were younger, had lower fasting plasma glucose during OGTT, and were diagnosed with GDM later in pregnancy (*p* < 0.05). The pregnancy outcomes were comparable between the groups, except for significantly lower macrosomia and large-for-gestational-age infants (LGA) frequency in Group A (*p* = 0.02 and *p* < 0.01, respectively).

The results of univariate and multivariate multinomial logistic regression are summarized in Table 2. The following variables were included in the models: 1-h PPG < 140 mg/dL criterion, mode of treatment, maternal age, prepregnancy maternal BMI, OGTT results, gestational age at diagnosis, and gestational age at delivery (only for macrosomia). In univariate regression analysis, 1-h PPG < 140 mg/dL criterion was correlated with increased odds ratios of macrosomia (*p* < 0.05) and LGA (*p* < 0.01). 1-h PPG < 140 mg/dL criterion was associated with significantly higher rates of macrosomia and LGA in all multivariable multinomial logistic regression models except for ‘Model 3 for macrosomia’. Moreover, odds ratios for macrosomia in all models were significantly increased with a higher prepregnancy BMI (Table 2).

**Table 2:** Models of logistic regression for macrosomia and LGA in GDM patients

Predictor	Univariate		Model 1^a^		Model 2^a^		Model 3^b^	
	OR (95% CI)	*p* value	OR (95% CI)	*p* value	OR (95% CI)	*p* value	OR (95% CI)	*p* value
*Macrosomia*								
1-h PPG < 140 mg/dL	2.674 (1.082–6.607)	**0.033**	3.573 (1.263–10.112)	**0.016**	3.305 (1.099–9.943)	**0.033**	2.952 (0.941–9.265)	0.064
Insulin treatment	1.302 (0.639–2.651)	0.4672	1.706 (0.791–3.680)	0.174	1.668 (0.746–3.821)	0.209	1.377 (0.588–3.226)	0.462
Maternal age (per 1 year increase)	1.052 (0.974–1.136)	0.1956	0.999 (0.916–1.090)	0.983	0.739 (0.927–1.112)	0.739	1.005 (0.913–1.106)	0.917
Prepregnancy BMI (per 1 kg/m^2^ increase)	1.026 (0.998–1.055)	0.065	1.034 (1.002–1.068)	**0.036**	1.042 (1.006–1.079)	**0.023**	1.041 (1.005–1.078)	**0.024**
OGTT at 0′ (per 10 mg/dL increase)	1.196 (0.875–1.635)	0.2608					1.186 (0.743–1.894)	0.475
OGTT at 120′ (per 10 mg/dL increase)	0.931 (0.688–1.259)	0.6413					0.901 (0.635–1.278)	0.557
Gestational age at delivery (per 1 week increase)	1.307 (1.009–1.694)	**0.0428**						
Gestational age at diagnosis (per 1 week increase)	0.975 (0.923–1.029)	0.3574			0.984 (0.926–1.045)	0.592		

Predictor	**Univariate**		**Model 1**		**Model 2**		**Model 3**^**c**^	
	OR (95% CI)	*p* value	OR (95% CI)	*p* value	OR (95% CI)	*p* value	OR (95% CI)	*p* value
*LGA*								
1-h PPG < 140 mg/dL	4.946 (2.066–11.841)	**0.0003**	4.637 (1.835–11.719)	**0.001**	4.319 (1.657–11.258)	**0.003**	4.118 (1.554–10.908)	**0.004**
Insulin treatment	1.082 (1.016–1.152)	**0.0135**	1.628 (0.894–2.964)	0.111	1.389 (0.737–2.617)	0.31	1.234 (0.643–2.369)	0.527
Maternal age (per 1 year increase)	1.052 (0.974–1.136)	0.1956	1.04 (0.973–1.112)	0.246	1.05 (0.978–1.127)	0.176	1.039 (0.965–1.118)	0.308
Prepregnancy BMI (per 1 kg/m^2^ increase)	1 (0.968–1.032	0.9915	1.009 (0.973–1.046)	0.632	1.012 (0.975–1.050)	0.526	1.010 (0.972–1.049)	0.621
OGTT at 0′ (per 10 mg/dL increase)	1.166 (0.894–1.521)	0.2567					1.189 (0.826–1.712)	0.352
OGTT at 120′ (per 10 mg/dL increase)	1.083 (0.871–1.345)	0.4737					0.939 (0.731–1.207)	0.625
Gestational age at diagnosis (per 1 week increase)	0.998 (0.950–1.047)	0.9262			1.025 (0.972–1.081)	0.362		

^a^Adjusted for gestational age at delivery

^b^Adjusted for gestational age at delivery and gestational age at diagnosis

^c^Adjusted for gestational age at diagnosis

Values in bold type are statistically significant at *p* < 0.05

## Discussion

The main goal in GDM management is to maintain a normal glycemic profile during pregnancy in order to reduce (‘normalize’) the risk of perinatal complications. Multiple randomized controlled trials have shown that GDM treatment reduces rates of adverse pregnancy outcomes [1, 2]. However, the extent of this risk reduction remains unsatisfactory. Our results show that the use of less stringent criteria for 1-h PPG in GDM (i.e. < 140 mg/dL vs. < 120 mg/dL) markedly increases the rate of macrosomia and LGA (Table 2).

Furthermore, it is noteworthy that in our study only the 1-h PPG target criterion was an independent predictor for both LGA and macrosomia. However, as data from continuous glucose monitoring systems (CGM) show, asymptomatic episodes of hypoglycemia and mean glucose levels < 87 mg/dL are associated with an increased risk of small-for-gestational-age infants (SGA) [1, 2]. Yet, we have not observed a significant difference of the SGA incidence between Group A and Group B. Moreover, no significant differences between groups were found regarding the need for insulin therapy, the rates of cesarean section, or preterm delivery. In conclusion, lowering a threshold for blood glucose at 1 h after a meal to < 120 mg/dL in GDM patients reduces the risk of LGA and macrosomia in their offspring, without increased incidence of SGA. Therefore, it is effective and safe and does not generate additional costs for the healthcare system.

## Strengths and limitations

A major strength of this study is a relatively large cohort of clinically well-characterized Caucasian women with GDM and detailed information concerning pregnancy outcomes in both study groups. The potential limitation of this study is its single-center and retrospective design. However, it enabled us to ensure that all of the participants received the same diabetic care and that our results are in line with real-world outcomes. Nevertheless, we are unable to provide final, conclusive, and universal recommendations**.** Similarly to the HAPO study [3], we did not obtain data on gestational weight gain.

## Conclusions

Macrosomia and LGA were less frequent among offspring of GDM mothers, who followed tighter postprandial blood glucose threshold, without an increased prevalence of SGA. Postprandial treatment target of < 140 mg/dL is a significant independent predictor for both macrosomia and LGA in GDM patients. Lowering postprandial glycemic target to < 120 mg/dL appears to be clinically effective and safe approach to GDM management. In order to ameliorate pregnancy outcomes in GDM women, further well-designed, randomized and prospective studies are required.

## Data Availability

The datasets generated during and/or analyzed during the current study are available from the corresponding author on reasonable request.
